# Experimental Infections with *Mycoplasma agalactiae* Identify Key Factors Involved in Host-Colonization

**DOI:** 10.1371/journal.pone.0093970

**Published:** 2014-04-03

**Authors:** Eric Baranowski, Dominique Bergonier, Eveline Sagné, Marie-Claude Hygonenq, Patricia Ronsin, Xavier Berthelot, Christine Citti

**Affiliations:** 1 INRA, UMR 1225, IHAP, Toulouse, France; 2 Université de Toulouse, INP-ENVT, UMR 1225, IHAP, Toulouse, France; Miami University, United States of America

## Abstract

Mechanisms underlying pathogenic processes in mycoplasma infections are poorly understood, mainly because of limited sequence similarities with classical, bacterial virulence factors. Recently, large-scale transposon mutagenesis in the ruminant pathogen *Mycoplasma agalactiae* identified the NIF locus, including *nifS* and *nifU*, as essential for mycoplasma growth in cell culture, while dispensable in axenic media. To evaluate the importance of this locus *in vivo*, the infectivity of two knock-out mutants was tested upon experimental infection in the natural host. In this model, the parental PG2 strain was able to establish a systemic infection in lactating ewes, colonizing various body sites such as lymph nodes and the mammary gland, even when inoculated at low doses. In these PG2-infected ewes, we observed over the course of infection (i) the development of a specific antibody response and (ii) dynamic changes in expression of *M. agalactiae* surface variable proteins (Vpma), with multiple Vpma profiles co-existing in the same animal. In contrast and despite a sensitive model, none of the knock-out mutants were able to survive and colonize the host. The extreme avirulent phenotype of the two mutants was further supported by the absence of an IgG response in inoculated animals. The exact role of the NIF locus remains to be elucidated but these data demonstrate that it plays a key role in the infectious process of *M. agalactiae* and most likely of other pathogenic mycoplasma species as many carry closely related homologs.

## Introduction

The term “mycoplasma” designates a large group of bacteria whose common feature is the lack of a cell wall. These atypical organisms belong to the class *Mollicutes* that has evolved by massive losses of genetic material from Gram-positive ancestors with low G+C content [1]–[3]. Successive genome reductions have left current mycoplasmas with an amount of genetic information that is close to the minimal requirements for sustaining autonomous life [4], [5], explaining in part their parasitic life-style. This strict association is often damaging to the host and several mycoplasma species are successful pathogens, capable of establishing persistent infections and causing debilitating diseases in humans and a wide range of animals [1]. Since most mycoplasmas are able to replicate in axenic media, this particular group of bacteria includes some of the smallest and simplest life-forms known. Along with the concept of a minimal cell, they have offered an experimental platform to explore and create the first organism controlled by a chemically synthesized genome [6]. While these studies have greatly expanded our understanding of cell biology fundamentals, factors involved in mycoplasma-host interactions remain poorly understood.

Of the mycoplasmas species relevant to the veterinary field, *Mycoplasma agalactiae* is an attractive model to study the infectious process of mycoplasmas in the natural host. First, it is an important pathogen responsible for contagious agalactia (CA) in small ruminants, an economically important disease notifiable to the World Organization for Animal Health [7]–[9]. Secondly, recent genomic analysis revealed that large portion of its genome has undergone gene exchanges with members of the mycoides cluster [10]. Although phylogenetically distant from *M. agalactiae*, all members of this cluster are ruminant pathogens, several of which being also responsible of CA in small ruminants. Among the genes that were exchanged, several encode surface and membrane components that may contribute to the infectious process. Finally, an additional interest in studying *M. agalactiae* is its close phylogenetic and genetic proximity with *M. bovis* [10], a re-emerging pathogen of growing concern for the cattle industry that induces similar clinical manifestations, including mastitis, arthritis and pneumonia [11]. Both ruminant pathogens possess a family of related surface proteins which high-frequency variation in expression and structure modulate the mycoplasma surface composition and accessibility, providing a means for these organisms to rapidly adapt to various environmental changes. Phase and size variations have been shown to occur in vitro with a frequency estimated at 10^−2^ to 10^−5^ events/cell/generation [12]. The type strain of *M. agalactiae*, strain PG2, carries a simplified version of this family, which members are designated as Vpmas (Variable protein of *M. agalactiae*) and are encoded by six genes, *vpma* U to Z, occurring as a cluster on the chromosome. In a given cell, only one *vpma* gene is expressed while in the overall population the six products occur at different rates [10], [12], [13]. This intra-clonal variation is due to the presence of a unique promoter at the *vpma* locus that is alternatively placed via a cut and paste mechanism in front of silent *vpma* genes, resulting in ON and OFF switching in Vpma expression [12]. This phenomenon is known to occur *in vitro* although it has never been truly documented over the course of an infection, despite its potential role in host colonization and in escaping the host-immune system.

Whole genome sequencing of a number of mycoplasmas species failed to reveal similarities with virulence factors identified in more classical bacteria suggesting that these minimal pathogens have evolved unconventional strategies to interact with their hosts [1]. An exception includes a magnesium-dependent nuclease homologue to the staphylococcal SNase, which role in *M. agalactiae* host-survival or host-pathogenicity has still to be demonstrated [14]. New opportunities to investigate factors involved in mycoplasma-host interactions have emerged through the development of molecular tools for the genetic manipulation of mycoplasmas [15]–[17]. Recently, we reported the development of a cell culture assay for the high-throughput screening of mutants with reduced growth capacities upon co-culture with host cells [18], [19]. Using this approach, 62 genomic loci of *M. agalactiae* were identified out of a library of ca. 2000 mutants as contributing to this phenotype. Of the 62 loci potentially involved in *M. agalactiae* host-interaction, one was of particular interest because two mutants having inserted a transposon at different position of the same gene, *nifS*, displayed most extreme growth-deficient phenotype in cell culture [18]. This locus is composed of two genes, *nif*S and *nif*U that are co-transcribed [19] and encodes homologues of SufS and SufU, two proteins presumably involved in Fe-S cluster biosynthesis in Gram-positive bacteria [20].

While cell culture provides a simple and efficient screening system for genome-scale analysis of *M. agalactiae* loci involved in host-interaction, the expression and regulation of the corresponding biological functions are expected to be far more complex in the host context, raising the question of the *in vivo* relevance of pre-screening cell assays. In this study, we formally addressed this issue by defining the virulence of the NifS mutants in the natural host. The *in vivo* model we developed consisted in inoculating lactating ewes by the subcutaneous route with low doses of *M. agalactiae* and to monitor mycoplasmas shedding in milk over three weeks of infection. This model allowed us to evaluate the capacity of these mutants to spread from the point of inoculation to the mammary gland, and hence to address their ability to colonize, replicate and survive in the animal host when compared to the wild type. We also evaluated how the Vpma variations can contribute to the success of the infection and possibly influenced the outcomes of *in vivo* studies when using defined mutants. This was performed by following the dynamic of the Vpma expression over the course of infection. For this purpose, we monitored the Vpma phenotype of organisms sequentially recovered in milk and the corresponding humoral host response.

The inability of the two NifS mutants to generate a successful infection *in vivo* indicates that key functions contributing to *M. agalactiae* survival and dissemination were affected and will be discussed. This study also brings into a new light the dynamic changes in mycoplasma surface composition that occurs over the course of the infection by showing the rapid in-host oscillation in *M. agalactiae* Vpma expression, with multiple profiles co-existing in the same animal.

## Materials and Methods

### Bacterial Strains and Culture Conditions

**Figure 1:**
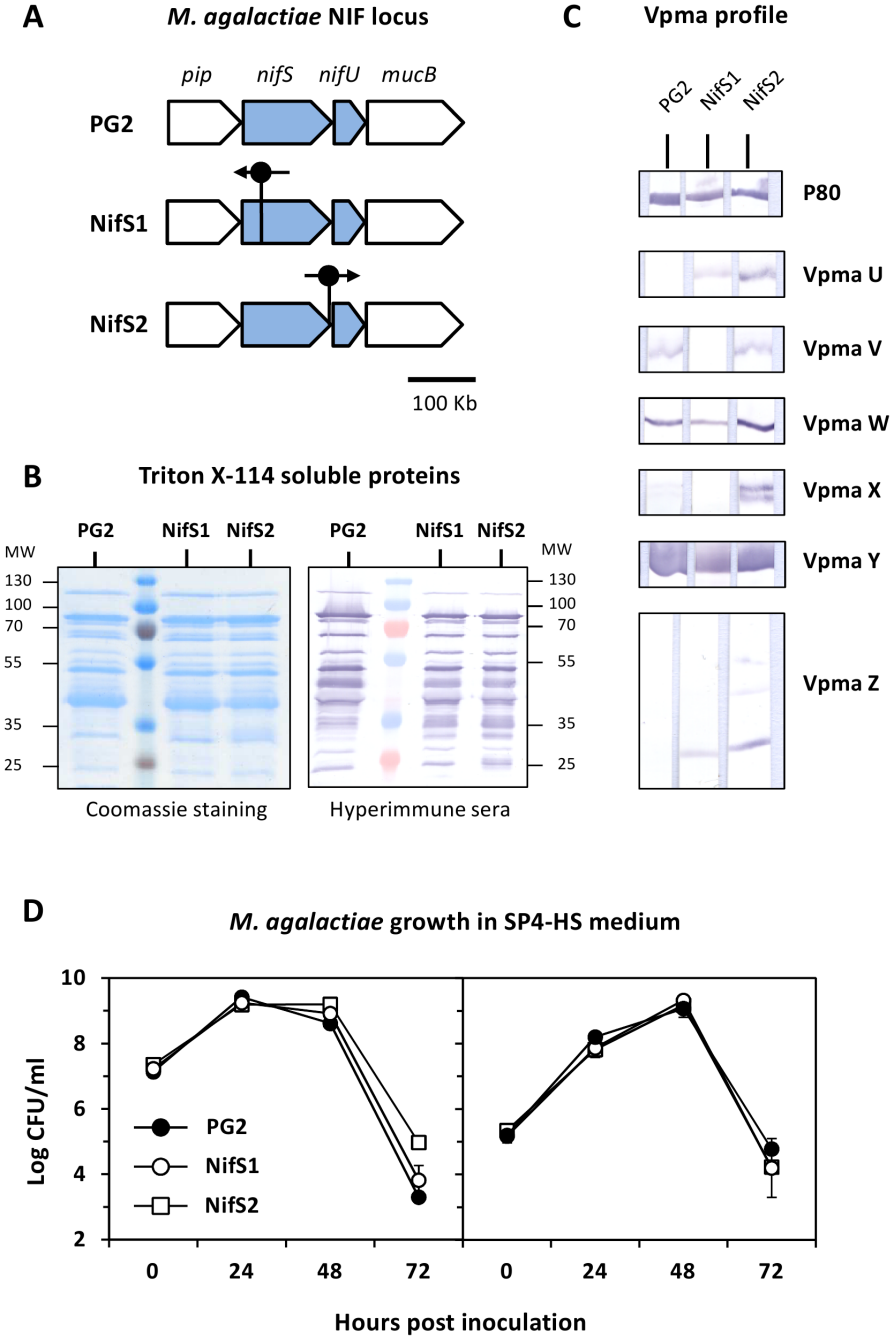
Genetic and antigenic features of *M. agalactiae* strain PG2 and NifS mutants used for animal inoculation. Knock-out mutants NifS1 and NifS2 were generated by transposon mutagenesis in *M. agalactiae* reference strain PG2 [18]. (**A**) Drawing illustrating the NIF locus (grey arrows) in *M. agalactiae*. Genes are represented according to their orientations on the genome. Transposon insertion sites in NifS mutants are indicated by a vertical bar with a closed circle, an arrow indicate its orientation. The insertion of the transposon in mutant NifS2 occurred within the stop codon of *nifS* [18]. The scale is indicated. (**B**) SDS-PAGE analysis of Triton X-114 soluble materials extracted from cultures of PG2 and NifS mutants followed by Coomassie staining or immunodetection using the PAL97 hyperimmune serum. Molecular weight standards (MW) are in kDa. (**C**) Characterization of phase variable, Vpma expression profile in *M. agalactiae* inoculums. Vpma lipoproteins U, V, W, X, Y and Z were detected using specific polyclonal antibodies (see Materials and Methods). A lipoprotein P80 antiserum was used as control. (**D**) Growth curves of PG2 and NifS mutants in SP4-HS medium. Mycoplasma titers used for inoculations were 10^7^ CFU/ml (left panel) and 10^5^ CFU/ml (right panel). The data are the means of three independent assays. Standard deviations are indicated by error bars.

*M. agalactiae* reference strain PG2 was used in this study [10]. Mutants NifS1 and NifS2, originally described as 7.82 and 7.134, were selected from a library of transposon knock-out mutants generated in strain PG2 [18]. They both carry a transposon in a genomic region designated as the NIF locus (CDS MAG0720 to MAG0730) (Fig. 1). The reference strain PG2 and the NifS mutants were cultured at 37°C in a modified SP4 medium, termed as SP4-HS, in which horse serum (HS) was substituted for fetal bovine serum. The SP4-HS medium was supplemented with 500 μg/ml cefalexin (Virbac). Mycoplasma cultures were stored at −80°C, and CFU titers were determined by serial dilutions in Dubelco’s phosphate-buffered saline (DPBS; Invitrogen) supplemented with 1% heat inactivated HS (Invitrogen). Dilutions were spotted (10 μl) onto solid SP4-HS medium and mycoplasma colonies were counted after 2 to 5 days incubation at 37°C. The absence of contaminating wild-type sequences in culture stocks of NifS mutants was tested by PCR amplification, as previously described [18].

### Ethics Statement

Experimentations were conducted as prescribed by the guidelines of the EU Council on Animal Care (86/609/CEE) and approved by the ethics committee of Midi-Pyrénées (France) under the agreement number MP/02/49/09/11. Animals were euthanized at day 28 post-inoculation by an intravenous injection of pentobarbital (140 mg/kg) (EUTHASOL VET, LE VET, Netherlands).

### Animals and Experimental Infection

Lactating ewes of the Lacaune breed, originating from a CA free area located in the south-west of France (Aveyron), were used for experimentations. Lactating ewes and their progeny were allocated into five experimental groups (5 ewes per group) and housed in separate rooms at animal facilities of the ENVT. Animals were shown to be free of *M. agalactiae* and other ruminant mycoplasma species, and exhibited undetectable levels of specific antibodies against *M. agalactiae*. Serological analyses were performed by ELISA (IDEXX *M. agalactiae* Screening Ab Test) and Western blot analysis (see below).

For inoculum preparations, *M. agalactiae* reference strain PG2 and NifS mutants were grown in SP4-HS medium. After 48 hours at 37°C, mycoplasma cultures were stored at −80°C as 1 ml aliquots. Before inoculation, frozen aliquots of mycoplasma cultures were thawed and CFUs were enumerated. On inoculation day, aliquots from the same batch were thawed and adjusted to the required CFU concentration by dilution in SP4-HS medium. Samples were kept on ice before inoculation. Each animal received a 2 ml subcutaneous injection of the inoculum or SP4-HS medium at the left shoulder. The doses of viable mycoplasmas injected to each experimental group were determined by titration of the inoculum before and immediately after animal inoculation. PCR amplifications failed to detect wild-type sequences in cultures of the NifS mutants, ruling out any possible contamination with either the parental strain or other mutants (data not shown).

### Clinical Examination and Sample Collection

Animals were examined and clinical signs were recorded and scored from day 7 before inoculation to day 28 post-inoculation (PI), with two- or three-days intervals. Milk and whole-blood samples were collected at regular interval throughout the experiments. Milk production was determined twice a week. Lymph nodes were examined and collected at necropsy (day 28), and stored at −80°C.

### Bacteriological and PCR Examination of Biological Samples

After collection, milk and whole-blood samples (collected in EDTA tubes) were diluted 1∶10 and 1∶100 in 2 ml SP4-HS medium (containing 500 μg/ml cefalexin and 0.01% thallium acetate). Three days following incubation at 37°C, cultures were distributed in 96-well plates (Falcon) and spotted onto solid SP4-HS medium (containing 500 μg/ml cefalexin and 0.01% thallium acetate) using a 96-pin replicator (Boekel Scientific). The volume of the sample transferred by one pin of the replicator was estimated at about 1 μl. The development of mycoplasma colonies was observed after 7 to 14 days incubation at 37°C. The minimal amount of detectable CFU was estimated at 10^2^ CFU (5×10^2^ CFU/ml) by testing serial dilutions of a mycoplasma culture stock.

Lymph nodes were separated from the surrounding fat, homogenized manually using sterile blades and resuspended in 2 ml SP4-HS medium. After 4 h incubation at 37°C, supernatants were diluted 1∶1 and 1∶20 in 2 ml SP4-HS medium, and the remaining suspensions were stored at −80°C. After 3 days incubation at 37°C, primary cultures were sub-cultured under the same conditions (dilution 1∶10). Primary cultures and sub-cultures were tested for the presence of mycoplasmas as described above.

Mycoplasma cultures were further examined by PCR amplification. Samples (1.5 ml) were centrifuged at 12,000×g for 20 min. The bacterial pellet was resuspended in 50 μl lysis buffer (0.1 M Tris-HCl, pH 8.5; 0.05% Tween 20; 0.25 mg/ml proteinase K), incubated at 37°C for 1 h followed by proteinase K inactivation at 95°C for 10 min. PCR amplifications (50 μl) were performed as described previously [21] using 2 μl of lysates and the *M. agalactiae*-specific primers MAPol-1F (5′-CATTGAACCTCTTATGTCATTTACTTTG-3′) and MAPol-5R_mod (5′-CTATGTCATCAG-CTTTTGAGTGA-3′), which span genomic positions 78626 to 78653, and 78866 to 78888, respectively.

### SDS-PAGE and Western Blot Analysis of *M. agalactiae* Triton X-114 Extracts

Mycoplasmas grown in SP4-HS medium were collected by centrifugation at 10,000×g and resuspended in DPBS (Invitrogen). Protein concentration was determined using the Quick Start Bradford protein assay (Bio-Rad). Triton-X114 soluble proteins were extracted with 1/9 volume of Triton X-114 solution (10 mM Tris-HCl pH 7.4; 150 mM NaCl; 10% Triton X-114). After 1 h incubation at 4°C under mild agitation, aqueous and detergent phases were separated by incubation at 37°C. After 5 min centrifugation at 12,000×g, the detergent phase was found as an oily droplet at the bottom of the tube. The detergent phase containing most hydrophobic, membrane proteins was washed 3 times with 9 volumes of Tris-buffered saline (TBS) (10 mM Tris-HCl pH 7.4; 150 mM NaCl), 1 h incubation at 4°C and phase separation at 37°C. Triton X-114 was removed by protein precipitation in methanol. Proteins resuspended in Laemmli sample buffer were separated by SDS-PAGE electrophoresis using the Mini-Protean electrophoresis system (Bio-Rad). Proteins were visualized by staining using the Bio-Safe Coomassie stain (BioRad). For Western blotting, proteins were transferred to Protran nitrocellulose membranes (Whatman). The amount of Triton X-114 soluble material transferred to membrane strips was equivalent to 10^8^ CFU of *M. agalactiae*. Membranes were blocked in TBS containing 5% skimmed-milk for 2 hours, then incubated overnight at 4°C with experimental sera diluted (1/125) in TBS containing 0.05% Tween 20 and 10% decomplemented horse serum (Invitrogen) or 1% skimmed milk. Western blots were developed with an HRP-labeled secondary antibody (DAKO; P0163) and 4-chloro-naphtol as substrate. The PAL97 hyperimmune serum was collected from a sheep naturally infected by *M. agalactiae*. The anti-P80 sheep serum, kindly provided by P. Giammarinaro, was produced by animal immunization with a P80 recombinant protein (data not shown). Vpma-specific polyclonal antibodies were used as previously described [22].

## Results

### Subcutaneous Inoculation with Low Doses of *M. agalactiae*: Colonization of the Udder and Shedding in Milk

Experimental models for ruminant mycoplasmoses are generally conducted with high infectious doses inoculated via the respiratory tract, the eye conjunctiva or the mammary gland. These parameters were not best suited for our study as we aimed at (i) monitoring the mycoplasma systemic colonization of the mammary gland from a different point of entry, (ii) recovering mycoplasmas during the course of infection without invasive procedures, and (iii) controlling the size of the inocula injected *de facto*. As well, we wanted to define whether low infectious dose can be used for future signature-tagged mutagenesis approaches, not described here.

Two experimental groups of lactating ewes were inoculated subcutaneously with 10^3^ or 10^5^ CFU of PG2 (experimental groups PG2-10^3^ and PG2-10^5^, respectively), while a control group received growth medium alone by the same route (Table 1). Animal behavior was monitored during 28 days and remained unchanged throughout this period, without any decrease in appetite or significant difference in rectal temperatures in between experimental and control groups (data not shown). Milk production remained stable in each group, with only two animals, animal A5 in group PG2-10^3^ and animal A8 in group PG2-10^5^, exhibiting an hypogalactia of the left udder-half (80% reduction in milk production). No other clinical sign was observed in infected and control animals. Subclinical mammary infections with *Escherichia coli* or coagulase-negative Staphylococci were identified in groups PG2-10^3^ (animals A4 and A5) and PG2-10^5^ (animals A6 and A8) (Table 1).

**Table 1:** Overview of the experimental infections with *M. agalactiae* strain PG2 and NifS mutants in lactating ewes using the subcutaneous route.

Animalgroup	Infectiousdose (CFUs)^a^	Hypo-galactia^b^	Milksamples^c^	Serologicalresponse^d^	Lymphnodes^e^	Otherbacteria^f^
Control	–	0/5	0/5	0/5	0/5	2/5
PG2-10^3^	3.2±1.0×10^3^	1/5	5/5	5/5	3/5	2/5
PG2-10^5^	2.3±0.2×10^5^	1/5	4/5	5/5	4/5	2/5
NIF1	1.6±0.2×10^5^	0/5	0/5	0/5	0/5	0/5
NIF2	1.6±0.3×10^5^	0/5	0/5	0/5	0/5	0/5

a Mean mycoplasma CFUs inoculated to each experimental group;

b number of animals with reduced milk production;

c number of animals with at least one milk sample found positive for *M. agalactiae*;

d number of animals that developed a serological response to *M. agalactiae*;

e number of animals with at least one lymph node found positive for *M. agalactiae* (the lymph nodes analyzed are indicated in Fig. 2);

f number of animals with subclinical mammary infections with *Escherichia coli* or coagulase-negative Staphylococci.

The capacity of *M. agalactiae* to infect its natural host and to colonize the mammary gland was investigated in milk samples collected at regular intervals from up to 28 days after inoculation. As shown in Table 2, all experimentally infected animals (except A7, see below) were found to excrete mycoplasmas in milk, while no mycoplasma was recovered from the control group. One exception was animal A7 in group PG2-10^5^, which remained negative for all samples tested. Two patterns of excretion clearly emerged in the experimental groups infected by PG2. One concerned a majority (8/10) of animals and was characterized by a transient excretion between days 18 and 28 post-inoculation (Table 2). The other was limited to one animal in each group, A5 in group PG2-10^3^ and A8 in group PG2-10^5^, and corresponded to a continuous excretion that started as early as 7 days post-inoculation and lasted until the end of the experiment. Mycoplasma excretion was mainly detected in a single udder-half in 21/27 positive samples. One exception was animal A4 in group PG2-10^3^, for which mycoplasmas were always isolated from both udder-halves (Table 2). Examination of whole blood samples collected at different times throughout the experiment failed to reveal the presence of mycoplasmas, with a detection limit estimated at 10^2^ CFU (5×10^2^ CFU/ml). Negative cultures of whole blood and milk samples were further analyzed by PCR amplification that confirmed the absence of mycoplasmas in these samples (data not shown). The presence of *M. agalactiae* in positive cultures was confirmed by PCR amplification using species-specific primers (data not shown).

**Table 2:** Mycoplasma excretion and serological response in animals inoculated with *M. agalactiae* strain PG2 and NifS mutants.

Biologicalassay	Animalgroup	Animal	Day post inoculation										
			0–4	7	9	11	14	16	18	21	23	25	28
		A1	−	−	−	−	−	−	−	−	R	−	−
		A2	−	−	−	−	−	−	−	R	−	−	−
	PG2-10^3^	A3	−	−	−	−	−	−	−	L	−	−	−
		A4	−	−	−	−	−	−	−	−	LR	LR	LR
		A5	−	−	−	L	L	L	L	L	LR	L	LR
Isolation of *M.*		A6	−	−	−	−	−	−	L	−	−	−	−
*agalactiae* in		A7	−	−	−	−	−	−	−	−	−	−	−
milk samples^a^	PG2-10^5^	A8	−	L	L	L	L	LR	L	R	L	L	L
		A9	−	−	−	−	−	−	−	−	−	L	−
		A10	−	−	−	−	−	−	−	−	−	L	−
	NIF1	A11 to A15	−	−	−	−	−	−	−	−	−	−	−
	NIF2	A16 to A20	−	−	−	−	−	−	−	−	−	−	−
		A1	−	−	−	+	+	+	+	+	+	+	+
		A2	−	−	−	−	−	−	−	+	+	+	+
	PG2-10^3^	A3	−	−	−	−	+	+	+	+	+	+	+
		A4	−	−	−	−	−	−	−	−	−	−	+
		A5	−	−	−	−	−	−	−	+	+	+	+
Serological IgG		A6	−	−	−	−	+	+	+	+	+	+	+
response^b^		A7	−	−	−	−	+	+	+	+	+	+	+
	PG2-10^5^	A8	−	−	+	+	+	+	+	+	+	+	+
		A9	−	−	+	+	+	+	+	+	+	+	+
		A10	−	−	−	−	+	+	+	+	+	+	+
	NIF1	A11 to A15	−	−	−	−	−	−	−	−	−	−	−
	NIF2	A16 to A20	−	−	−	−	−	−	−	−	−	−	−

a The excretion of *M. agalactiae* in the mammary gland is indicated with a letter indicating udder halves found positive: right (R), left (L) or both (LR);

b the detection of specific IgG antibodies to Triton X-114 soluble antigens of *M. agalactiae* in animal sera are indicated (+).

The dissemination and survival of *M. agalactiae* in lactating ewes was further investigated by examination of lymph node samples collected at necropsy (Fig. 2A). Here, PCR amplification was performed in parallel to cultivation in order to overcome the outgrowth by contaminating bacteria or fungi that occurred occasionally despite of the presence of cefalexin and thallium acetate in the medium. With the exception of three animals (A2, A3 and A6), all infected ewes displayed at least one positive lymph node, with a total of 80% of the samples (10/12) being PCR positive (Fig. 2A). Lymph nodes frequently found positive were the left prescapular, next to the site of inoculation (left shoulder) and the supramammary lymph nodes, (see Fig. 2A and Table 2). Nevertheless, the PCR positive iliac lymph node detected in animal A8 suggests a broader dissemination for *M. agalactiae*. These data indicated that *M. agalactiae* is able to survive within the host even for long periods, and most likely in the lymph nodes.

**Figure 2:**
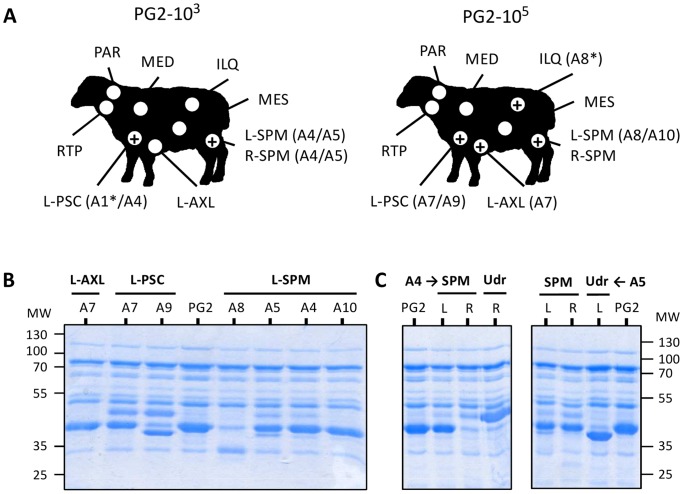
Recovery of *M. agalactiae* from the lymph nodes of lactating ewes experimentally infected by the subcutaneous route. (**A**) Schematic drawing illustrating the recovery of *M. agalactiae* from animal lymph nodes in experimental groups PG2-10^3^ and PG2-10^5^. Experimental groups were inoculated subcutaneously at the left shoulder with different doses of *M. agalactiae* strain PG2 (see Table 1). The lymph nodes collected at day-28 pi are indicated by an open circle (L-AXL: left-axillary; ILQ: Iliac; MED: mediastinal; MES: mesenteric; L-PSC: left-prescapular; L-SPM: left-supramammary; R-SPM: right-supramammary; RTP: retropharyngeal; PAR, parotid). For each group, lymph node samples found positives by PCR amplification are indicated (+) and the corresponding animals are given in parenthesis. *M. agalactiae* was isolated from all PCR positive samples, except those designated by an asterisk (*). (**B**) SDS-PAGE analysis of Triton X-114 soluble proteins of mycoplasmas recovered from animal lymph nodes in experimental groups PG2-10^3^ (animals A4 and A5) and PG2-10^5^ (animals A7, A8, A9 and A10). (**C**) Illustration of mycoplasma membrane protein diversification upon PG2 (PG2) dissemination in the animal host. Mycoplasmas were isolated from (L) left- and (R) right- retromammary (RTM) lymph nodes or udder-halves (Udr). Proteins were visualized by Coomassie staining after SDS-PAGE. Molecular weight standards (MW) are in kDa.

Altogether, these results indicate that high infectious doses are not required for successful infection and dissemination of *M. agalactiae* in the natural, ovine host. Thus, the experimental infection of lactating ewes by subcutaneous route and the subsequent recovery of mycoplasmas from milk samples provide a simple and non-invasive assay for *in vivo* screening of large pools of *M. agalactiae* mutants.

### Serological Response and Dynamics of Variable Vpma Surface Proteins

The development of a serological response in animals infected with *M. agalactiae* was monitored by Western blotting (Table 2). Despite a 100-fold difference in the dose used for animal inoculation, both experimental groups developed IgG antibodies to *M. agalactiae* surface antigens. Yet, the appearance of specific IgG in group PG2-10^3^ was delayed when compared to group PG2-10^5^.

**Figure 3:**
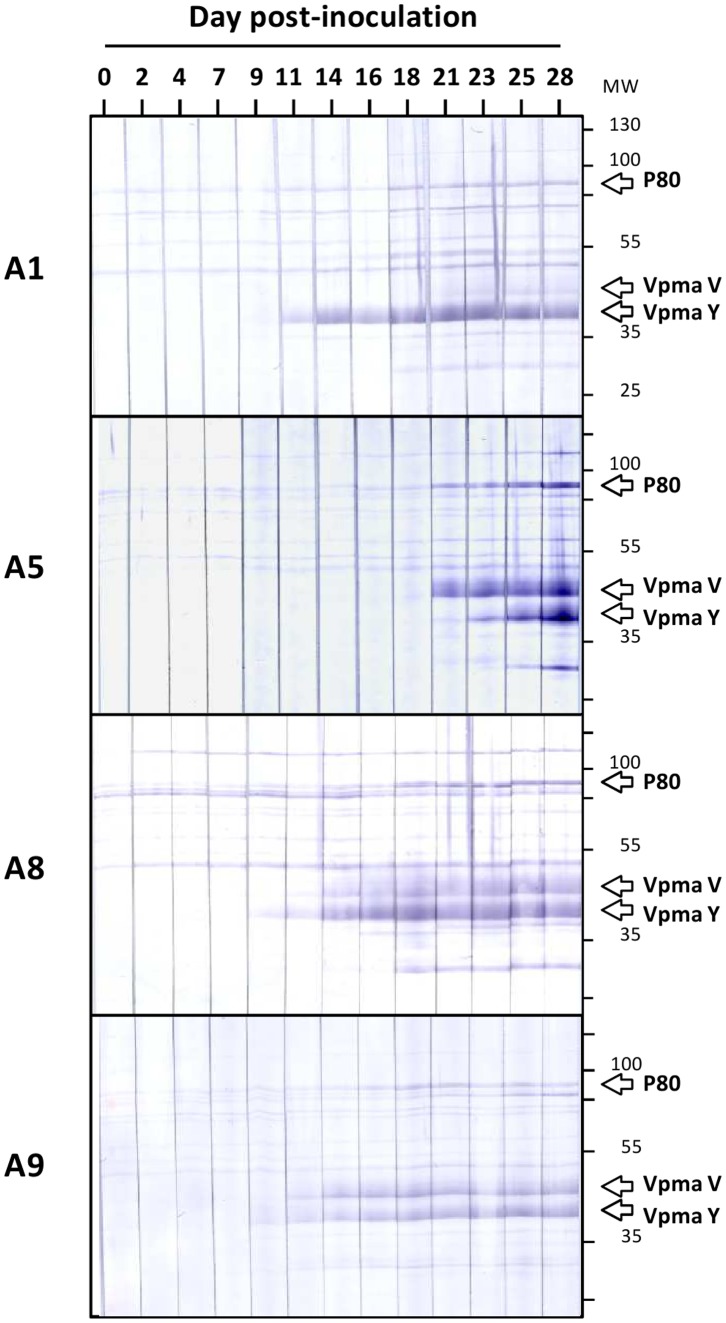
Development of a serological response to *M. agalactiae* in lactating ewes experimentally infected by the subcutaneous route. Immunoblotting pattern of *M. agalactiae* Triton X-114 soluble proteins obtained using sera from experimental groups PG2-10^3^ (animals A1 and A5) and PG2-10^5^ (animals A8 and A9) collected at different days post-inoculation (0 to 28). The position at which lipoprotein P80 (80 kDa) and phase variable Vpma lipoproteins V (44 kDa) and Y (39 kDa) migrate are indicated. Molecular weight standards (MW) are in kDa.

To better characterize the development of the humoral response within single animals, sera from animals A1, A5, A8 and A9, which exhibited a strong reactivity against *M. agalactiae*, were used. Sera collected at regular intervals throughout the experiment were analyzed by Western blotting using the Triton-X114 soluble proteins of strain PG2 that contain most hydrophobic, membrane proteins (Fig. 3). Immunoprofiles revealed differences among animals in terms of (i) the kinetic of the IgG response, with animals A1, A8 and A9 having a strong IgG response from day 11 while that in A5 appeared to be delayed and (ii) specificity, with the 4 animals displaying different immunoprofiles. A strong reactivity was mainly obtained with proteins migrating between 35 and 55 kDa (Fig. 3). More specifically, antibodies reacted strongly with two protein bands showing molecular weight values similar to Vpmas V (44 kDa) and Y (39 kDa), two phase variable lipoproteins of *M. agalactiae* (data not shown).

**Figure 4:**
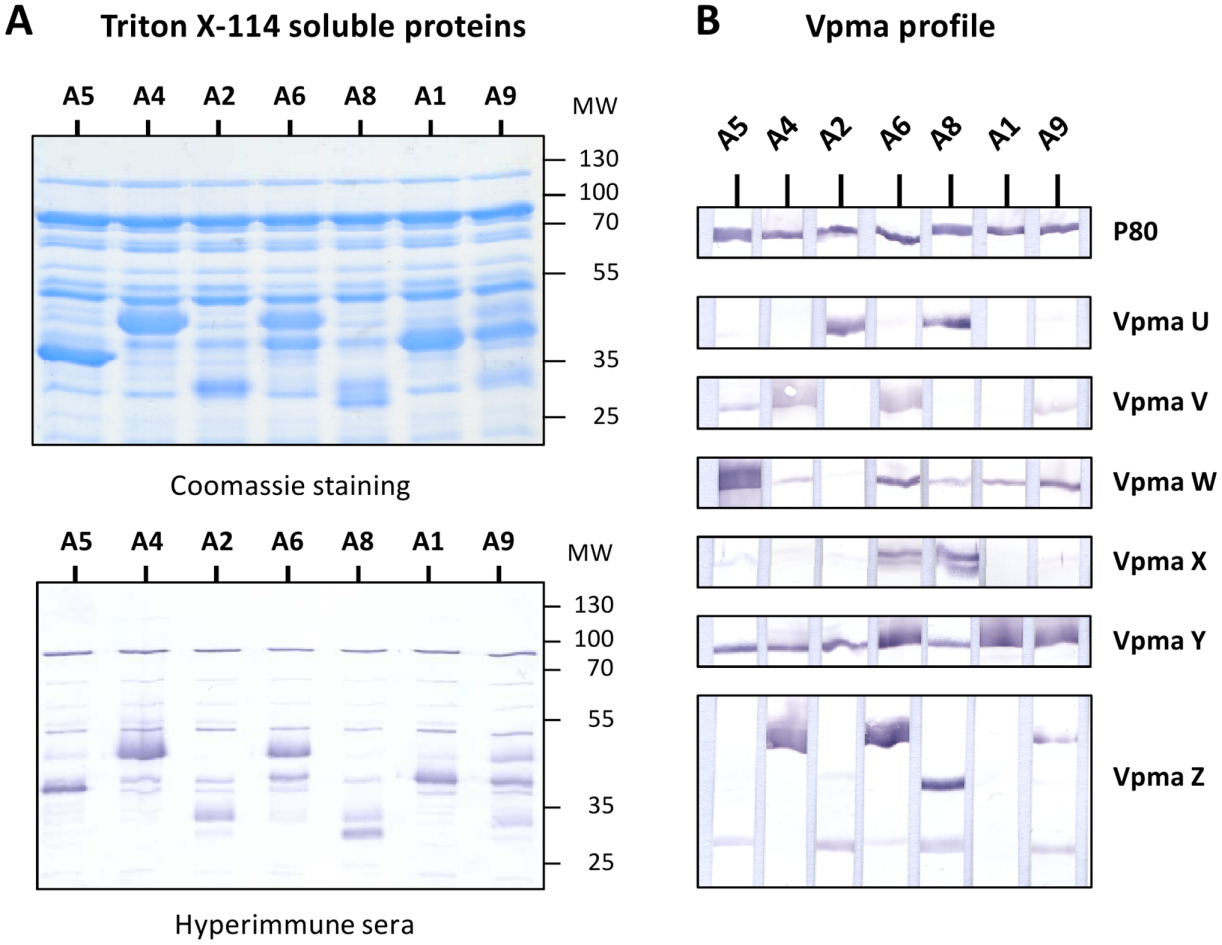
Diversification of *M. agalactiae* antigenic structure following a single passage in the animal host. (**A**) SDS-PAGE analysis of Triton X-114 soluble materials extracted from cultures of *M. agalactiae* recovered from milk samples at day 28 post-inoculation in experimental groups PG2-10^3^ (animals A1, A2, A4 and A5) and PG2-10^5^ (animals A6, A8 and A9) followed by Coomassie staining, immunodetection using the PAL97 hyperimmune serum, and (**B**) characterization of the Vpma expression profile using specific polyclonal antibodies (see Materials and Methods). A lipoprotein P80 antiserum was used as control. Molecular weight standards (MW) are in kDa.

Differences in the development of the humoral response to *M. agalactiae* prompted us to compare the antigenic profile of *M. agalactiae* isolated from milk samples. For this purpose, the Triton-X114 soluble proteins from seven mycoplasma-positive cultures, corresponding to animals positive at day 28 (A4, A5 and A8) or to animals that were positive once (A1, A2, A6 and A9), were analyzed by SDS-PAGE and Western blotting. As shown in Figure 4, their profile revealed major differences in protein expression. More specifically, these were associated to highly abundant proteins with molecular weight values that ranged between 35 and 55 kDa and that were predominantly recognized by the PAL97 hyperimmune serum (Fig. 4). These data suggested that *M. agalactiae* isolated from milk samples were expressing different Vpma lipoprotein profile and this was confirmed by using Vpma-specific polyclonal antibodies (Fig. 4).

While the PG2 inoculum expressed predominantly Vpma Y (Fig. 1), the Vpma profile exhibited by the seven isolates was highly heterogeneous, with some samples expressing predominantly Vpma W (A5), Vpma U (A2), Vpma V and Z (A4), or a complex mixture of several Vpmas reflecting the presence of several populations in the sample. A lipoprotein P80 antiserum was used as control and confirmed the constant expression of this lipoprotein in all the seven isolates. In contrast to *M. agalactiae* milk isolates, the Vpma expression profile exhibited by PG2 and NifS cultures used for animal inoculations were homogeneous (compare Vpma profiles in Fig. 1 and Fig. 4), apart from the inherent background in alternate Vpma populations and despite differences in their number of passages under laboratory conditions.

**Figure 5:**
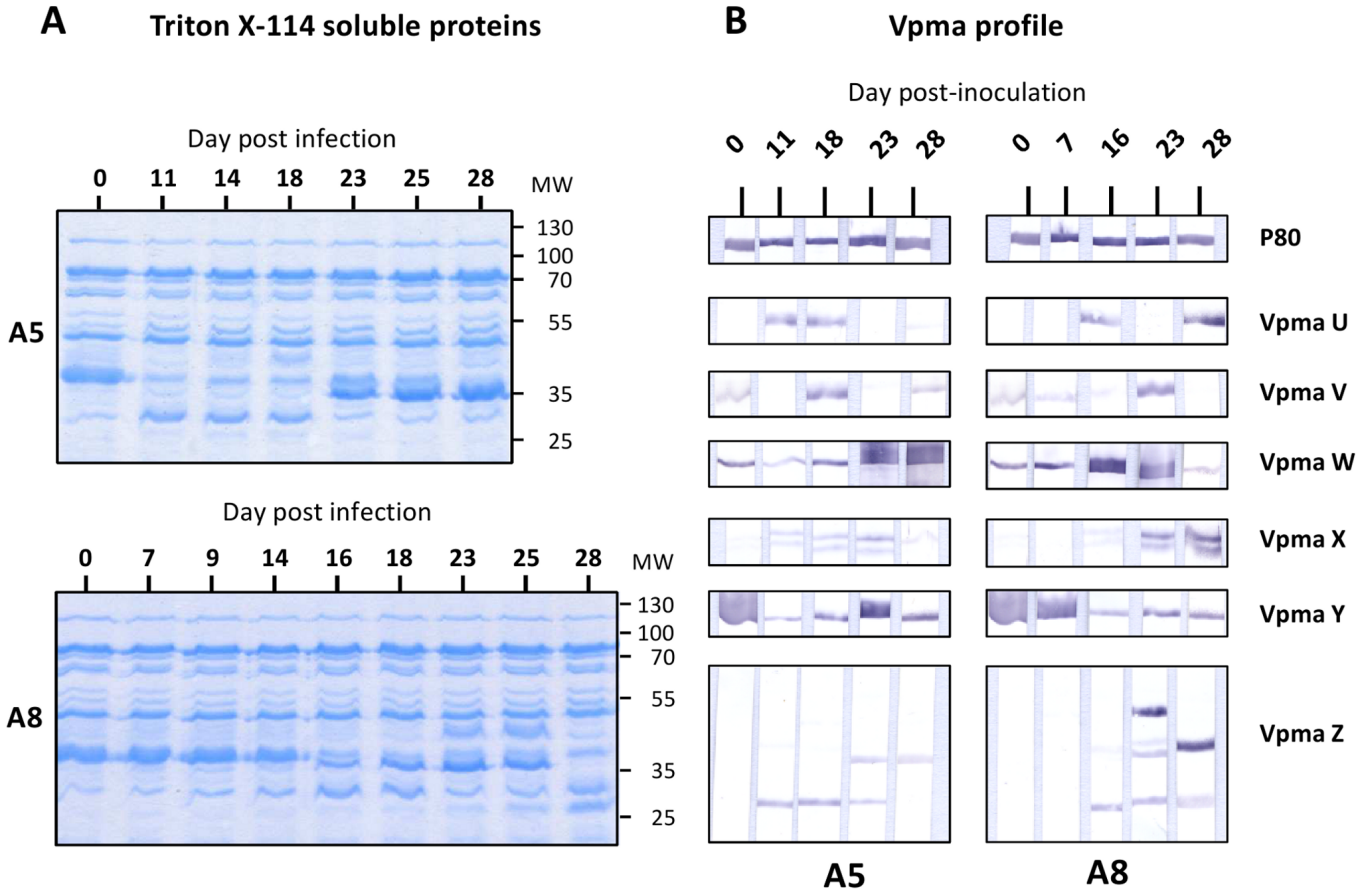
Stochastic changes in *M. agalactiae* antigenic structure upon colonization of the animal host. (**A**) SDS-PAGE analysis of Triton X-114 soluble proteins extracted from cultures of *M. agalactiae* recovered from milk samples collected from animals A5 and A8 at different days post-inoculation (0 to 28). (**B**) Characterization of the Vpma expression profile using specific polyclonal antibodies (see Materials and Methods). A lipoprotein P80 antiserum was used as control. Molecular weight standards (MW) are in kDa.

The long term excretion of mycoplasmas in the left half-udder of animals A5 and A8 allowed us to analyze changes in *M. agalactiae* Vpma profile over a period of 18 to 22 days. SDS-PAGE analysis of Triton-X114 soluble proteins from mycoplasma cultures indicated continuous changes in protein expression, and Vpma-specific polyclonal antibodies revealed a highly dynamic Vpma profile with stochastic changes in Vpma expression (Fig. 5).

To further investigate changes in the expression of *M. agalactiae* surface proteins upon replication *in vivo*, Triton-X114 soluble proteins of mycoplasmas recovered from lymph nodes were analyzed (Fig. 2B). As observed with milk isolates, important differences in protein expression profile were also found with mycoplasmas isolated from the axillar, prescapular and supramammary lymph nodes, suggesting that mycoplasma diversification is not restricted to the mammary gland, but can occur at different stage of the infection.

At day 28, mycoplasmas that circulated in animals A4 and A5 differed in their protein expression depending on whether they were isolated from the milk, or from the left- or right-supramammary lymph nodes (Fig. 2C), suggesting that diversification occurred independently in each environment.

Overall, these results indicate that (i) even a single passage *in vivo* can severely affect the *M. agalactiae* surface composition, (ii) several changes in Vpma expression may occur in the course of the infection in a single animal, and (iii) different Vpma profiles can be detected in different organs of the same animal.

### *M. agalactiae* Knock-out Mutants Selected in Cell Culture are Unable to Survive and Colonize the Ovine Host

Previous studies have identified the *M. agalactiae* NIF locus, composed of the co-transcribed *nifS* and *nifU* genes, as essential for mycoplasma growth in cell culture and suggested a possible role of this locus *in vivo* [18]. To formally address this issue, two groups of ewes, designated NIF1 and NIF2, were inoculated with 10^5^ CFU of the NifS1 and NifS2 mutants respectively (Table 1). Each of these mutants derived from the PG2 type strain used above and harbored a stable-transposon inserted at different positions in *nifS* but exhibited identical growth-deficient phenotypes upon co-cultivation with mammalian cells [18], while displaying wild-type growth in axenic conditions (Fig. 1D). Complementation studies previously confirmed the role of the NIF locus for *M. agalactiae* survival in cell culture and restored the wild-type phenotype [18]. Yet, complemented strains were not used in our study to avoid the use of a constant antibiotic treatment of the animals that would be required to preventing the rapid loss of extra-chromosomal plasmid carrying the wild-type genes. Instead, we hypothesized that the use of the two mutants in two independent groups, if resulting in the same outcome, would confirm the role of the NIF locus *in vivo*.

Biological samples from experimental groups NIF1 and NIF2 were collected and examined as for PG2 groups. In contrast to the two groups inoculated with PG2, attempts to isolate mycoplasmas from the milk of animals of groups NIF1 and NIF2 failed. No isolate was obtained from blood, as for PG2 groups. All animals remained negative throughout the experiment with a detection limit of our assay estimated at 10^2^ CFU (5×10^2^ CFU/ml). Negative cultures were further tested by PCR amplification that confirmed the absence of mycoplasma material in the samples tested. At necropsy, all lymph nodes tested were also found negative for mycoplasma (Table 1).

The inability of the NifS mutants to survive and colonize the host was further confirmed by the analysis of IgG antibody response that remained undetectable in the serum of all inoculated animals (Table 2). The antigenic profile of the two mutants was closely similar to that of the parental strain PG2, when using the PAL97 hyperimmune serum collected from a sheep naturally infected by *M. agalactiae* (Fig. 1). This excluded the possibility of a major change in the antigenic structure of the NifS mutants that could account for the lack of IgG detection. This was further confirmed by using Vpma-specific polyclonal antibodies that identified Vpma Y as the main phase variable surface lipoprotein expressed by each population, with subtle differences in Vpma lipoproteins expressed at background levels (Fig. 1). Finally, the absence of specific IgG antibodies in animals from groups NIF1 and NIF2 was further confirmed by Western blotting experiments carried out using NifS1 and NifS2 surface antigens, instead of PG2 materials (data not shown).

These results clearly demonstrate the essential role played by the NIF locus in *M. agalactiae* survival and colonization of the ovine host. They also demonstrate that cell culture provide a useful strategy for the pre-screening of large libraries of *M. agalactiae* mutants.

## Discussion

The avirulent phenotype exhibited by two knock-out mutants harboring a transposon inserted at different positions of the single-copy NifS gene (MAG0720) indicate that a key virulence factor of *M. agalactiae* maps within the NIF locus (MAG0720 and MAG0730). In contrast to the parent PG2 strain, *M. agalactiae* NIF mutants were unable to establish an infection in the ovine host as no mycoplasma nor specific antibodies were detected over the course of the experiment in either of the two groups infected by one or the other mutants. It is thus unlikely that other genetic alterations could be responsible for the avirulence of the two NifS mutants, since these were identified independently based on their phenotype in cell culture and have a transposon inserted at different position of the same gene, while deriving from the same PG2 type strain.

The almost strict conservation of the NIF locus among all mycoplasma genomes sequenced so far emphasizes its biological importance (Table S1), and raises questions regarding the role of this particular locus during the infectious process. As expected, the highest level of similarity was observed for the closely related *M. bovis*, suggesting that CA in small ruminants can provide an interesting model to study *M. bovis* infection in cattle. In *M. agalactiae*, the NIF locus consists of two CDSs encoding homologues of nitrogen fixation proteins NifS and NifU, two proteins involved in iron-sulfur Fe-S cluster biogenesis. Fe-S clusters are ubiquitous and versatile prosthetic groups found in enzymes catalyzing a variety of redox reactions or proteins serving as redox sensors in a broad range of regulatory processes [23]. Bacteria use three distinct systems for Fe-S cluster biogenesis: ISC, SUF, and NIF machineries. Initially annotated as NIF homologues, sequence features of the SUF machinery were clearly identified in *M. agalactiae* NifS and NifU proteins that were indicative of a cysteine desulfurase (SufS) and a protein scaffold (SufU), respectively [18]. In contrast to many bacteria, SUF is the unique Fe-S biogenesis system in most *Firmicutes* and in mycobacteria [20], [24]. Interestingly, SUF is associated with stress tolerance in many bacteria by ensuring the assembly of Fe-S proteins under conditions of iron limitation and oxidative stress, and has been also linked to virulence in a few pathogenic species [25], [26]. Therefore, it is tempting to speculate that these two proteins might play an essential role in *M. agalactiae* survival within the host by their contribution in the biosynthesis of iron-sulfur clusters. However, given the multiple biological functions involving cysteine desulfurases and scaffold proteins [27], further studies are needed to define precisely the contribution of the NIF locus in the infectious process of *M. agalactiae*. The absence of an IgG response in animals inoculated with the NifS mutants suggests a critical role of the NIF locus in the initial steps of colonization by the mycoplasma. This hypothesis is consistent with the rapid loss of viability of the two NifS mutants upon co-culture with mammalian cells [18].

Proteins involved in metabolic regulation or stress response in bacteria can have additional biological functions and some are now regarded as virulence factors [28]. This capacity of some bacterial proteins to exhibit more than one function, a concept known as protein moonlighting, is highly appealing in the context of the reduced mycoplasma coding capacity. Studies with several mycoplasma species, including the human pathogen *M. pneumoniae*, have identified moonlighting functions in several glycolytic enzymes and chaperones including the glyceraldehydes-3-phospahate dehydrogenase, the pyruvate dehydrogenase subunit B, proteins with enolase activity, and the elongation factor Tu [28]–[33]. The moonlighting function associated with these proteins is conditioned by their location at the mycoplasma surface [28]. Whether multifunctional enzymes, such as proteins encoded by the NIF locus, may have a moonlighting activity in mycoplasmas remains highly speculative, but our study clearly demonstrate a role for housekeeping genes in *M. agalactiae* virulence.

While experimental models of infection with *M. agalactiae* were developed using elevated doses that ranged from 10^5^ to 10^9^ CFUs [34]–[38], the model validated in the present study suggests that even minimal infectious doses, as low as 10^3^ CFU, can establish infection in lactating ewes and disseminate to various body sites including lymph nodes and the mammary gland. This greatly expands the number of mutants that can be tested simultaneously in a single animal, and provide a useful system for *in vivo* functional studies using large pools of mutants in a signature-tagged mutagenesis like strategy.

A major drawback to performing genetic screens in cell culture is the absence of host immune defenses. To secure their survival, mycoplasmas have evolved sophisticated mechanisms of phase and antigenic variation [12]. Phase variation have been shown to occur *in vitro* with a high frequency (10^−2^ to 10^−5^ events/cell/generation) resulting in highly heterogeneous populations. It is assumed that high-frequency phase and size variation of surface components in Mollicutes that colonize immunocompetent hosts largely contributes to escaping the host immune response. Our results confirmed the rapid modification of *M. agalactiae* surface architecture upon replication *in vivo*, which can occur even after a single passage in the animal host, and identified Vpmas surface lipoproteins as major contributors of antigenic variation in the natural host. The remarkable plasticity of *M. agalactiae* was further documented by the diversity of the antigenic profiles identified that differed considerably, not only between animals but also between samples in the same animal, with different profiles coexisting at the same time. *M. agalactiae* Vpma profile in udder-halves of animals exhibiting a continuous excretion profile was characterized by important shifts in expression, providing the first detailed description of sequential changes affecting the surface architecture of this pathogen during the course of infection in its natural host. It would be interesting to determine if these shifts are consecutive to a re-infection of these animals by antigenic variants excreted by other animals in the group. This may have important implications for the circulation and persistence of *M. agalactiae* within the herd.

The role of surface antigenic variation in the pathogenesis of mycoplasma infections is a recurrent question. Infection studies with the avian pathogen *M. gallisepticum* showed that antigenic variation is detectable within a few days after infection, before the onset of a serological response [39]. While surface antigenic variation may facilitate infection by *M. gallisepticum*, a recent study with *M. agalactiae* phase-locked mutants expressing only a single Vpma surface lipoprotein suggested that Vpma phase variation was not necessary for establishing an infection in the ovine host, but might critically influence the survival and persistence of the pathogen under natural field conditions [38]. In the present study, PG2 infected animals exhibited marked differences in the kinetic of mycoplasma excretion in milk and the development of the serological response. While all PG2 inoculated animals developed specific IgG antibodies, the onset of the response varied considerably among animals. Although the number of animals was low, it is noteworthy that animals A4, A5 and A8, which were all characterized by a prolonged mycoplasma excretion, developed a serological response only after the beginning of the excretion, while animals that were characterized by a single excretion day exhibited a specific IgG response prior to mycoplasma detection in milk samples. Further studies involving larger experimental groups are needed to evaluate the influence of the serological response against *M. agalactiae* on the duration of mycoplasma excretion in the ovine host. This result, together with the rapid diversification of Vpma expression profile upon *M. agalactiae* replication in the animal host, raise important questions regarding the influence of these variations on functional studies involving infections with knock-out mutants. These results underscore the importance of characterizing the Vpma profile of knock-out mutants.

The avirulent phenotype exhibited by the NifS mutants suggests that key functions contributing to *M. agalactiae* survival and dissemination in the ovine host can be identified upon genetic screening in cell culture, providing a convenient system for high-throughput analysis of large transposon mutant libraries and the study of virulence factors in a large number of pathogenic mycoplasma species. Host-colonization by mycoplasmas is a valuable criterion to compare the infectivity of knock-out mutants *in vivo*, a way to overcome some limitations linked to individual susceptibility and to combine genetic screens in cell culture with *in vivo* studies. Finally, *M. agalactiae* replicating in the ovine host may also provide valuable information regarding *Mycoplasma bovis*-host interaction, given the genetic proximity of these two pathogenic species.

## Supporting Information

Table S1 Conservation of *M. agalactiae* NifS among main pathogenic mycoplasma species.
(DOC)
