# Correction: Experimental Infections with *Mycoplasma agalactiae* Identify Key Factors Involved in Host-Colonization

**DOI:** 10.1371/journal.pone.0103158

**Published:** 2014-07-16

**Authors:** The PLOS ONE Staff

Table 2 appears incorrectly due to errors that occurred during the typesetting process. The authors have provided a corrected version of Table 2 to improve readability.

**Table 2:** Mycoplasma excretion and serological response in animals inoculated with *M. agalactiae* strain PG2 and NifS mutants.

Isolation of *M. agalactiae* in milk samples a											
	Day post inoculation										
Animal (group)	0-4	7	9	11	14	16	18	21	23	25	28
A1 (PG2-10^3^)	-	-	-	-	-	-	-	-	R	-	-
A2 (PG2-10^3^)	-	-	-	-	-	-	-	R	-	-	-
A3 (PG2-10^3^)	-	-	-	-	-	-	-	L	-	-	-
A4 (PG2-10^3^)	-	-	-	-	-	-	-	-	LR	LR	LR
A5 (PG2-10^3^)	-	-	-	L	L	L	L	L	LR	L	LR
A6 (PG2-10^5^)	-	-	-	-	-	-	L	-	-	-	-
A7 (PG2-10^5^)	-	-	-	-	-	-	-	-	-	-	-
A8 (PG2-10^5^)	-	L	L	L	L	LR	L	R	L	L	L
A9 (PG2-10^5^)	-	-	-	-	-	-	-	-	-	L	-
A10 (PG2-10^5^)	-	-	-	-	-	-	-	-	-	L	-
A11 to A15 (NIF1)	-	-	-	-	-	-	-	-	-	-	-
A16 to A20 (NIF2)	-	-	-	-	-	-	-	-	-	-	-

Serological IgG response ^b^											
	Day post inoculation										
Animal (group)	0-4	7	9	11	14	16	18	21	23	25	28
A1 (PG2-10^3^)	-	-	-	+	+	+	+	+	+	+	+
A2 (PG2-10^3^)	-	-	-	-	-	-	-	+	+	+	+
A3 (PG2-10^3^)	-	-	-	-	+	+	+	+	+	+	+
A4 (PG2-10^3^)	-	-	-	-	-	-	-	-	-	-	+
A5 (PG2-10^3^)	-	-	-	-	-	-	-	+	+	+	+
A6 (PG2-10^5^)	-	-	-	-	+	+	+	+	+	+	+
A7 (PG2-10^5^)	-	-	-	-	+	+	+	+	+	+	+
A8 (PG2-10^5^)	-	-	+	+	+	+	+	+	+	+	+
A9 (PG2-10^5^)	-	-	+	+	+	+	+	+	+	+	+
A10 (PG2-10^5^)	-	-	-	-	+	+	+	+	+	+	+
A11 to A15 (NIF1)	-	-	-	-	-	-	-	-	-	-	-
A16 to A20 (NIF2)	-	-	-	-	-	-	-	-	-	-	-

a The excretion of *M. agalactiae* in the mammary gland is indicated with a letter indicating udder halves found positive: right (R), left (L) or both (LR); ^b^ the detection of specific IgG antibodies to Triton X-114 soluble antigens of *M. agalactiae* in animal sera are indicated (+).
