# Supplementary material for: Experimental Infections with Mycoplasma agalactiae Identify Key Factors Involved in Host-Colonization
Source: PLoS One. 2014 Apr 3;9(4):e93970. doi: 10.1371/journal.pone.0093970 (PMC3974822; doi:10.1371/journal.pone.0093970)
Supplement: Table S1 — Conservation of M. agalactiae NifS among main pathogenic mycoplasma species. (DOC) [file pone.0093970.s001.doc]

**Table S1.** Conservation of *M. agalactiae* NifS among main pathogenic mycoplasma species.

| **Species (strain)** | **Group ^a^** | **Host** | **CDS** | **Length** | **Identity ^b^** | **Similarity ^b^** |
| --- | --- | --- | --- | --- | --- | --- |
| *M. agalactiae* (PG2) | Hominis | Ovine, caprine | MAG0720 | 386 | 100% | 100% |
| *M. bovis* (PG45) | Hominis | Bovine | MBOVPG45_0081 | 386 | 79% | 89% |
| *M. synoviae* (53) | Hominis | Avian | MS53_0322 | 388 | 46% | 66% |
| *M. hominis* (PG21) | Hominis | Human | MHO_1230 | 389 | 48% | 66% |
| *M. pulmonis* (UAB CTIP) ^c^ | Hominis | Rodent | MYPU_1730 | 387 | 46% | 66% |
| *M. hyorhinis* (HUB-1) | Hominis | Swine | MHR_0305 | 383 | 45% | 64% |
| *M. conjunctivae* (HRC/581T) | Hominis | Ovine | MCJ_002850 | 380 | 44% | 63% |
| *M. hyopneumoniae* (232) | Hominis | Swine | mhp217 | 381 | 43% | 64% |
| *M. pulmonis* (UAB CTIP) ^c^ | Hominis | Rodent | MYPU_1720 | 393 | 37% | 57% |
| *M. capricolum* sp. *capricolum* (ATCC 27343) | Spiroplasma | caprine | MCAP0469 | 412 | 33% | 54% |
| *M. mycoides* sp. *capri* (95010) | Spiroplasma | caprine | MLC_4810 | 412 | 33% | 54% |
| *M. leachii* (PG50) | Spiroplasma | Bovine | MSB_A0482 | 412 | 33% | 54% |
| *M. gallisepticum* (R) | Pneumoniae | Avian | MGA_1128 | 400 | 35% | 56% |
| *M. iowae* (695) | Pneumoniae | Avian | GUU_04901 | 405 | 33% | 54% |
| *M. genitalium* (G37) | Pneumoniae | Human | MG336 | 408 | 34% | 50% |
| *M. pneumoniae* (M129) | Pneumoniae | Human | MPN487 | 408 | 33% | 49% |

^a^ Phylogenetic group; ^b^ Similarity searches were carried out using Blastp (E value cutoff: e-8) against database found in Molligen 3.0 (<http://cbib1.cbib.u-bordeaux2.fr/molligen3b/>); ^c^ The genome of *M. pulmonis* strain UAB CTIP includes two copies of *nifS*.
